# Comprehensive Exome Analysis of Immunocompetent Metastatic Head and Neck Cancer Models Reveals Patient Relevant Landscapes

**DOI:** 10.3390/cancers12102935

**Published:** 2020-10-12

**Authors:** Hui Li, Hoi-Lam Ngan, Yuchen Liu, Helen Hoi Yin Chan, Peony Hiu Yan Poon, Chun Kit Yeung, Yibing Peng, Wai Yip Lam, Benjamin Xiaoyi Li, Yukai He, Vivian Wai Yan Lui

**Affiliations:** 1 School of Biomedical Sciences, Faculty of Medicine, The Chinese University of Hong Kong, Hong Kong SAR, China; lihui@link.cuhk.edu.hk (H.L.); 1155098201@link.cuhk.edu.hk (H.-L.N.); yuchenliu@cuhk.edu.hk (Y.L.); helenchanhy@link.cuhk.edu.hk (H.H.Y.C.); peonyphy@cuhk.edu.hk (P.H.Y.P.); yeungchunkit@cuhk.edu.hk (C.K.Y.); 2 Department of Medicine, Medical College of Georgia, Augusta University, Augusta, GA 30912, USA; YPENG@augusta.edu (Y.P.); yhe@augusta.edu (Y.H.); 3 Lee’s Pharmaceutical (HK) Limited, Hong Kong Science Park, Hong Kong SAR, China; wy.lam@leespharm.com (W.Y.L.); drli@leespharm.com (B.X.L.)

**Keywords:** AT-84, SCC VII, whole-exome sequencing analysis, utility analyses, 5 immunocompetent metastatic HNC models

## Abstract

**Simple Summary:**

Head and neck cancer (HNC), once metastasized, is very difficult to treat as there are limited therapy options. Animal models of such can be very useful for preclinical drug development, including precision medicine and immunotherapy. By whole-exome analyses and functional annotation of five commonly used immune-intact metastatic models for HNC research, we aimed to fully annotate their genomic profiles on key cancer genes as well as signaling-, metastasis-, immune- and drug-related events, with direct comparisons with those of patient tumors. Our analyses revealed marked genomic similarities between these models and HNC patient tumors, and identified new potential drug targets for metastatic HNC. We suggest that more of such immune-relevant metastatic HNC models should be developed with full genomic annotations in order to enable preclinical research, and to accelerate precision medicine and immunotherapy development for this devastating cancer.

**Abstract:**

Immunocompetent metastatic head and neck cancer (HNC) models, although scarce, can help understanding cancer progression and therapy responses in vivo. Their comprehensive genome characterizations are essential for translational research. We first exome-sequenced the two most widely used spontaneous metastatic immunocompetent models, namely AT-84 and SCC VII, followed by comprehensive genomic analyses with three prior-sequenced models (MOC2, MOC2-10, and 4MOSC2), together with patient tumors for utility assessment. AT-84 and SCC VII bear high HNC tumor resemblance regarding mutational signatures—*Trp53*, Fanconi anemia, and MAPK and PI3K pathway defects. Collectively, the five models harbor genetic aberrations across 10 cancer hallmarks and 14 signaling pathways and machineries (metabolic, epigenetic, immune evasion), to extents similar in patients. Immune defects in *HLA-A* (*H2-Q10*, *H2-Q4*, *H2-Q7*, and *H2-K1*), *Pdcd1*, *Tgfb1*, *Il2ra*, *Il12a*, *Cd40*, and *Tnfrsf14* are identified. Invasion/metastatic genome analyses first highlight potential druggable *ERBB4* and *KRAS* mutations, for advanced/metastatic oral cavity cancer, as well as known metastasis players (*Muc5ac*, *Trem3*, *Trp53*, and *Ttn*) frequently captured by all models. Notable immunotherapy and precision druggable targets (*Pdcd1*, *Erbb4*, *Fgfr1*, *H*/*Kras*, *Jak1*, and *Map2k2*) and three druggable hubs (RTK family, MAPK, and DNA repair pathways) are frequently represented by these models. Immunocompetent metastatic HNC models are worth developing to address therapy- and invasion/metastasis-related questions in host immunity contexts.

## 1. Introduction

Head and neck cancer (HNC) is the sixth most common cancer worldwide, with nearly 0.83 million new incidences and 0.43 million deaths per year (2018, International Agency for Research on Cancer, IARC) [1]. Its highly aggressive nature remains the major clinical challenge. At diagnosis, over 50% of patients have advanced disease with loco-regional spread or metastases [2]. These patients often have high mortality with median survival of less than one year [3,4]. Thus, a deeper understanding of HNC biology and underlying disease progression will accelerate the development of a more effective therapy for invasive/metastatic HNC.

Among pan-cancers characterized by The Cancer Genome Atlas (TCGA), HNC has been revealed as an immune-altered cancer [5,6]. Emerging evidences are revealing the importance of immunity in HNC progression, invasion, metastasis, and drug responses. Studies in other cancers have begun to uncover intricate interplays between patient tumor genomic aberrations and the host immunity, such as cytokine-mediated cancer cell growth, invasion and metastasis, cancer drug-induced immunogenic cell death, and, recently, the ability of tumor-specific mutations and neo-antigens in the active shaping of tumor immune microenvironment, etc. [7,8,9,10,11,12,13,14]. In HNC, previous studies on immune regulators, for instance, the transforming growth factor-β (TGF-β) [15], interleukin-10 (IL-10) [16], and immune checkpoint molecules programmed cell death protein 1 (PD-1), and programmed death-ligand 1, also known as PD-L1 [17], and to a lesser extent metastatic gene functions [18], have all utilized immunocompetent mouse models for detailed functional investigations and generated important knowledge, advancing our understanding of HNC tumorigenesis and progression in immune-related contexts. Recently, one immunogenomics bioinformatic study has also taken the advantages of engineering immunocompetent HNC mouse models to prove a novel immunoactivating and CD8^+^ T cell-inviting tumor microenvironment in HNC shaped by MAPK pathway mutations, which was first informed by bioinformatics [19]. In terms of metastasis, although not many immunocompetent metastatic HNC models have been established, studies in other cancers are showing great promises of immunocompetent models for the effective identification of metastasis gene targets for potential therapy developments. These include the identification of *KDELR3*, *BMI*, *ANXA2*, *CCN*, *TYRP2*, *VLA-4*, *HSP70*, and *HSP90* genes for metastatic melanoma [20,21,22,23,24]; *SERPINE2*, *S100A9*, *TGM2*, *KLF4*, and *POEM* genes for breast cancer metastasis [25,26,27,28]; and *VEGFA*, *CCR6*, *PDGFR-β*, *CRAMP*, *RANKL*, and *IGF-IR* for metastatic lung cancer [29,30,31,32,33].

As of today, for HNC, there are five convenient xenograftable immunocompetent metastatic models available to help in addressing tumorigenesis, invasion/metastasis-related questions, as well as drug-induced or even precision medicine-induced effects in a host immunity context. These are AT-84 [34], SCC VII [35] (C3H background), MOC2 [36], MOC2-10 [36], and 4MOSC2 [17] (C57BL/6 background). Knowing the precise metastatic, targetable, and immune-related genomic profiles of these currently available models and the patient relevance of their genomic profiles will help researchers to fully utilize these models for both basic and translational research. Among these five models, AT-84 and SCC VII of C3H background are the two most widely used spontaneous metastatic immunocompetent models, but they have not been genomically characterized previously. Here, we first exome-profiled both AT-84 and SCC VII, followed by comprehensive analyses of all plausible metastatic and immunogenomic events of all five models. We then vigorously determined their utilities, thus human relevance, by direct comparison with TCGA-HNC patient tumor profiles. Our findings for AT-84 and SCC VII of C3H background demonstrate their high resemblance to HNC patient tumors, in capturing diverse mutational signatures relevant to HNC tumorigenesis, and key gene copy increases including *Pik3ca*, *Rptor*, and *Ctcf*. Importantly, mutational profile analyses of all five models show genetic defects on 10 cancer hallmarks and 14 HNC-relevant signaling pathways and machineries (including epigenetic regulatory, metabolic regulatory, and immune evasion machineries) to extents similar to HNC patients (TCGA). Immune-event analyses reveal patient-relevant aberrations of *HLA-A* (*H2-Q10*, *H2-Q4*, *H2-Q7*, and *H2-K1*), *Pdcd1*, *Tgfb1*, *Il2ra*, *Il12a*, *Cd40*, *Tnfrsf14,* and several checkpoint regulators. Invasion/metastatic genome analyses first highlight potential druggable *ERBB4* and *KRAS* mutations, present in 5.3% of metastatic HNC patients, that are commonly captured by these models. Strikingly, *Muc5ac*, *Trem3*, *Trp53*, *and Ttn*, known metastasis players in other cancers, are consistently mutated in all five metastatic models. For drugging purposes, notable immunotherapy and precision druggable targets (*Pdcd1*, *Erbb4*, *Fgfr1*, *Hras*, *Kras*, *Jak1*, and *Map2k2*) and three druggable hubs (EGFR family, MAPK, and DNA repair pathways) are most frequently represented by the models. In conclusion, more metastatic models should be developed to further advance HNC studies in immunocompetent contexts, in terms of metastasis, drugging, and tumor-immune interactions.

## 2. Results

### 2.1. Exome Characterization of AT-84 and SCC VII

AT-84 and SCC VII represent the two earliest established and most widely used xenograftable immunocompetent metastatic HNC models [34,35]. As of today, they have not been genomically characterized, limiting their utilization for potential precision medicine, invasion/metastasis targeting as well as tumor immunology studies. Both models were spontaneously derived in C3H background with metastatic abilities reminiscent of highly aggressive cancers in patients. SCC VII invades to the mylohyoid musculature, mandible, cervical lymph node, lung, and brain, and can cause cachexia as noted in advanced patients [37,38,39,40], while AT-84, when orthotopically inoculated in the oral site, can metastasize to the lung as noted in very advanced HNC patients [41,42].

**Figure 1:**
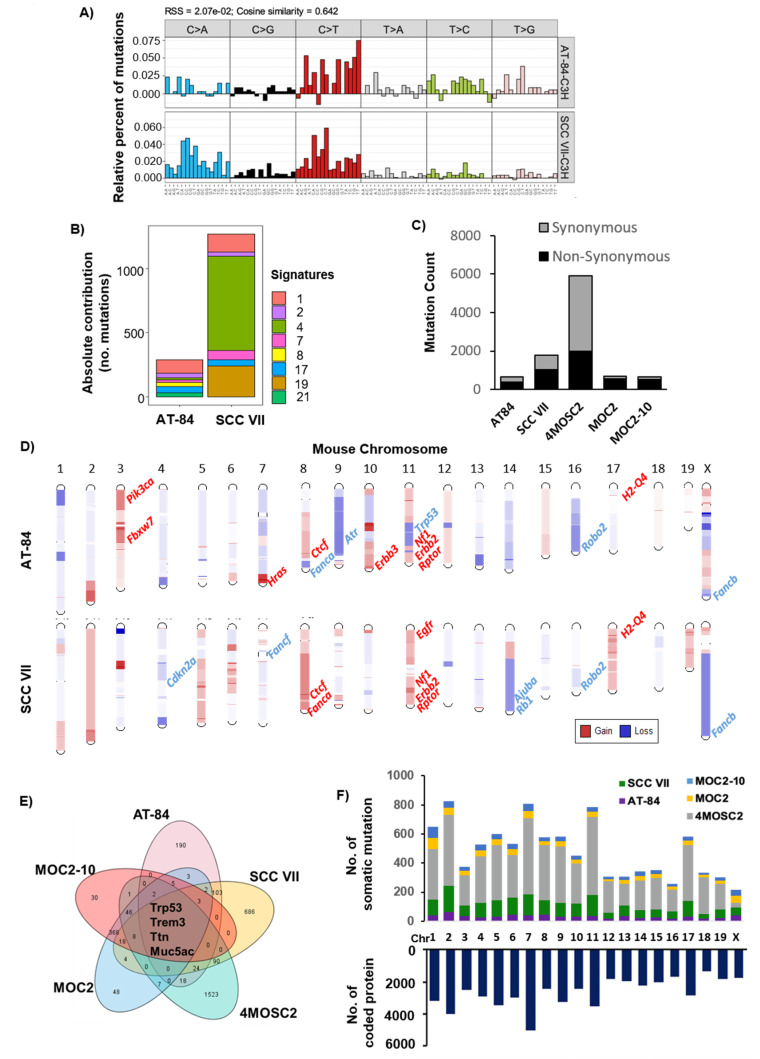
Comprehensive characterization of mouse head and neck cancer (HNC) models. (**A**) Mutational spectrum of all base substitutions of AT-84 and SCC VII. Different substitutional types are indicated in the vertical axes. (**B**) Mutation signatures of AT-84 and SCC VII. Bar plot showing the contribution of Catalogue of Somatic Mutations in Cancer (COSMIC) mutational signatures to reconstructing 96 mutational profiles. Color-coded columns represent different COSMIC signatures according to the legend on the right. (**C**) Bar plots showing mutation rates of AT-84, SCC VII, MOC2, MOC2-10, and 4MOSC2, respectively. The total number of synonymous and non-synonymous mutations are shown in grey and black bars, respectively. (**D**) Chromosomal gains (red) and losses (blue) in AT-84 and SCC VII. Some key HNC tumor-related genes are labeled. (**E**) Venn diagram showing overlapping somatic non-synonymous mutations among 5 mouse models, with numbers indicating somatic non-synonymous mutation number in each model. (**F**) Distribution of mutational events across mouse chromosomes with different colors representing events from different models (upper panel). The numbers of coding proteins in each mouse chromosome are shown (lower panel).

Here, we performed whole-exome sequencing at ~50× depth for both models, and found both models carrying predominant C>T transitions (Figure 1A), which have been reported to be common in HNC patient tumors [43]. Subsequent mutational signature analysis revealed HNC-relevant pathogenic signatures [44,45], including *APOBEC* edit (signature 2), tobacco smoke (signature 4), UV damage (signature 7), and aging-related signature (signature 1) (Figure 1B). Notably, SCC VII carried a dominating signature 21, indicative of a clear microsatellite instability (MSI) or genomically unstable phenotype. This finding was consistent with an apparently higher mutation rate of SCC VII (1786 somatic mutations with 1013 being non-synonymous mutations) vs. AT-84 (643 somatic mutations with 378 being non-synonymous mutations) (Figure 1C; Table S1).

Somatic copy number variations (CNVs) were determined by CNVkit. Both AT-84 and SCC VII have arm-level recurrent gains of mouse chr.2q, 8q, and 11q as well as a loss of 4q (Figure 1D). It has to be reminded that mouse chromosome and human chromosome numberings are not interchangeable, as gene orthologs reside on different chromosomes in mouse and human. Notably, chr.8q and 11q gains in both models represent gains of a highly HNC-relevant transcriptional repressor *Ctcf* (8qD3, a master regulator of the genome, human *CTCF*) [46], *Erbb2* (11qD, human *ERBB2*), *Rptor* (11qE2, an mTOC1 regulatory subunit, human *RAPTOR*) [47], and *H2-Q4* (17qB1, histocompatibility 2, Q region locus 4, human *HLA-A*). Common gene copy losses represented by both models with HNC patient relevance are *Robo2*, an important candidate tumor suppressor of the ROBO–SLIT pathway (16qC3), and *Fancb* gene (XqF5, human *FANCB*) involved in the Fanconi anemia (FA) pathway, which is altered in subsets of HNC patients. Figure S1 shows that the rates of occurrence of human *ROBO2* and *FANCB* gene copy losses in HNC tumors from TCGA-HNC Firehose (*N* = 510) are 70% (356/510) and 29% (150/510 cases), respectively (www.cbioportal.org [48,49]).

Specifically, gene copy gains of *Pik3ca*, *Hras*, and *Fbxw7*, all relevant to HNC tumors, are captured by AT-84, whereas copy gain of *Egfr* is captured by SCC VII. In HNC patient tumors, *EGFR*, *PIK3CA*, *HRAS*, and *FBXW7* are gained or amplified in 42% (213/510), 71% (363/510), 13% (64/510), and 7% (36/510), respectively (Figure S1). Notably, HNC-relevant gene copy losses of *Cdkn2a*, *Fancf*, *Ajuba*, and *Rb1* are all found in SCC VII, and *Atr* loss is found in AT-84. In HNC tumors, human *CDKN2A*, *FANCF*, *AJUBA*, *RB1*, and *ATR* homozygous and heterozygous losses are 59% (303/510), 27% (139/510), 15% (76/510), 41% (211/510), and 3% (16/510), respectively (Figure S1). In brief, AT-84 and SCC VII do harbor CNVs that are of HNC relevance, representing a sizeable number of HNC patient tumor CNVs. The comprehensive CNVs of AT-84 and SCC VII are listed in Table S2, while CNV data of the prior-sequenced MOC2, MOC2-10, and 4MOSC2 cell lines were not publicly available for analysis [17,36].

### 2.2. Cancer Gene Analysis Revealed Resemblance with Human HNC

AT-84 and SCC VII were derived from C3H background [34,35], whereas 4MOSC2, MOC2, and MOC2-10 were derived from C57BL/6 background [17,36]. Note that MOC2-10 is a subline of MOC2, thus it bears a highly similar mutational rate to that of MOC-2 [36,50]. Among all 5 lines, 4MOSC2 has the highest number of non-synonymous mutations, followed by SCC VII, MOC2, MOC2-10, and AT-84 (Figure 1C). Notably, across all 5 models, mutations of *Trp53*, *Trem3*, *Muc5ac*, and *Ttn*, corresponding to human *TP53*, *TREM-1/2*, *MUC5AC*, and *TTN*, are noted (Figure 1E). Assessing all mouse chromosomes, chr.2, 7, and 11 appeared to harbor the highest number of total mutational events (both synonymous and non-synonymous) as these three chromosomes have, on average, the highest number of protein-coding genes, as shown in Figure 1F.

**Figure 2:**
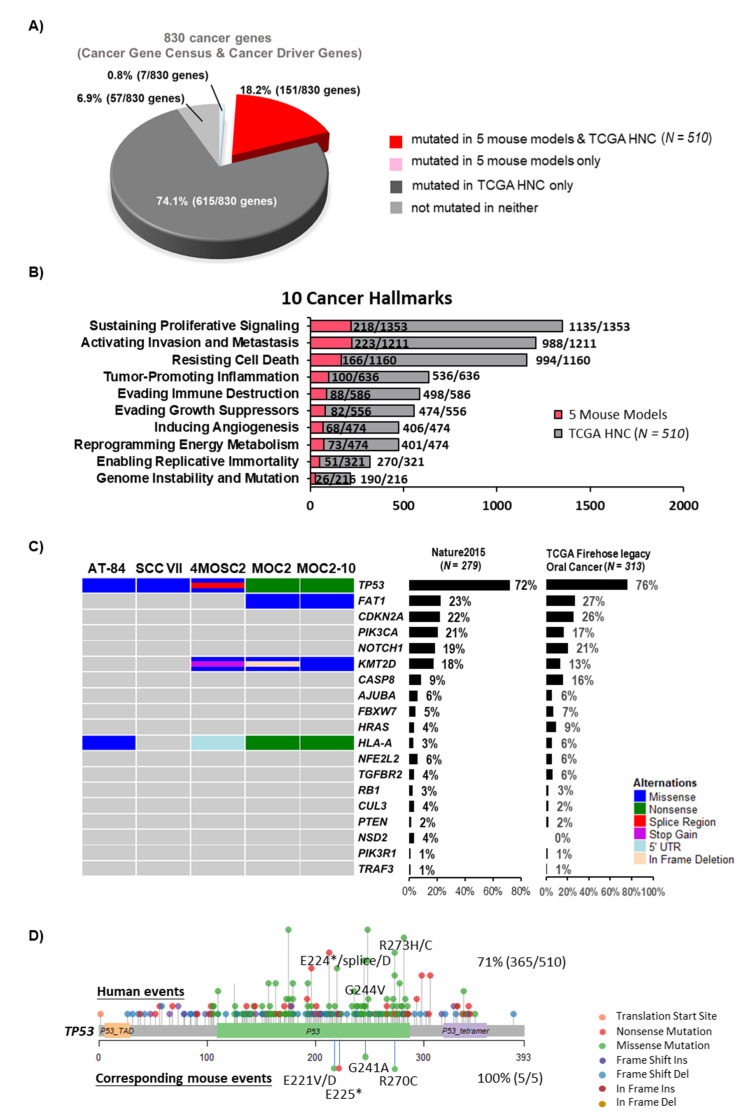
Mutations in mouse models show a high resemblance to human cancer. (**A**) A pie chart showing the recapitulation of cancer-related somatic mutations (830 genes) in 5 mouse cell lines vs. that of The Cancer Genome Atlas (TCGA)-HNC patient tumors (*N* = 510). Cancer-related genes with or without alterations in 5 mouse cell lines or HNC patient tumors are indicated according to the legend at the bottom. (**B**) Bar graph showing the somatic mutation counts of 10 cancer hallmark gene sets harbored by 5 mouse cell lines and TCGA-HNC (*N* = 510). The mutation counts of HNC patients are represented by grey bars, whereas those of mouse models are represented by red bars. (**C**) Oncoprints for 5 mouse models showing genes with statistically significantly mutated (MutSig, Q value < 0.1), with event frequencies in TCGA-HNC, all patient tumors, as well as TCGA-HNC oral tumors indicated with percentages on the right. (**D**) Mapping of mutation sites of *Trp53* mutations in 5 mouse models and human *TP53* hotspot mutations based on whole-exome sequencing data of TCGA-HNC (*N* = 510). TCGA: The Cancer Genome Atlas.

Cancer gene mutations deserve the highest priorities for biology and targeting studies. We thus determined the potential utility of all five models vigorously by comparing their cancer gene mutational events with those of HNC patient tumors using the most updated TCGA-HNC Firehose cohort (*N* = 510, www.cbioportal.org). We first assembled a combined human cancer gene list of 830 genes (assembled from the most recent TCGA pan-cancer study [51] and the COSMIC Cancer Gene Census), followed by conversion to mouse gene names for the ease of human gene name referencing (Table S3). A total of 766/830 cancer genes (93.1%) are found mutated in HNC patient tumors (151 and 615 cancer genes, indicated in red and deep grey, respectively; Figure 2A). Although not many, these 5 mouse models did capture mutational changes of 151/766 of HNC-relevant cancer genes (indicated in red, Figure 2A). Altered cancer genes represented by these mouse models include the human equivalent genes *TP53*, *BRCA2*, *HLA-A*, *PTPRB/C/K*, *KRAS*, *ROBO2*, *FGFR1*, *CTCF*, *JAK1/3*, *ERBB4*, *KMT2A*, and *KMT2D*, which have been biologically studied in human HNC, although their effects on immune-relevant contexts have not been investigated. Furthermore, more than half (94/148) of the remaining cancer gene events captured by these five models have not been previously studied in HNC (Table S4). Thus, these models provide unique opportunities for future investigation of these cancer gene mutations in HNC.

Zhang et al. [52] have recently defined cancer hallmark gene sets, highlighting key genes involved in each of the 10 cancer hallmarks. We then performed side-by-side comparisons of aberrations of 10 cancer hallmark gene sets in TCGA-HNC tumors and these 5 mouse models. A total of 458 hallmark genes (1637 non-synonymous mutations) are mutated in 5 mouse models across hallmarks of “sustaining proliferative signaling”, “activating invasion and metastasis”, “tumor-promoting inflammation”, “evading immune destruction”, “resisting cell death”, “enabling replicative immortality”, “evading growth suppressors”, “reprogramming energy metabolism”, “inducing angiogenesis”, and “genome instability and mutation” (Figure 2B). Human HNC tumors from TCGA revealed the highest mutation counts in 3 cancer hallmarks, namely “sustaining proliferative signaling”, “activating invasion and metastasis”, and “resisting cell death” (Figure 2B). Importantly, mutational counts of these top 3 hallmark events are largely similar, in proportion, to these 5 mouse models as compared to HNC patient tumors. In fact, the notable high rate of mutations found in gene sets for the “activating invasion and metastasis” hallmark appears to be consistent with the metastatic natures of these models.

Previous exome characterization study of HNC by TCGA identified 19 significantly mutated genes (by MutSig on 279 patient tumors, cutoff of q < 0.1) [53]. The mutation rates of these significantly mutated genes are updated by us using the most recent TCGA-HNC tumors, Firehose (*N* = 510 tumors; www.cbioportal.org) (Figure 2C). Since most of these mouse models (AT-84, 4MOSC2, MOC2, and MOC2-10) and over 60% of the cases of TCGA-HNC patient tumors were derived from the oral cavity, we also displayed the relative abundancies of these genetic events in oral cancer only (*N* = 308 cases). Note that the overall pattern of genetic events between all HNC cases and oral cancer only are largely similar. As shown in Figure 2C, we found that these 5 models did capture genetic aberrations of 4 of the 19 significantly mutated genes (mouse counterparts). These are *TP53* (*Trp53*), *FAT1* (*Fat1*), *KMT2D* (*Kmt2d*) and *HLA-A* (*H2-Q10*, *H2-K1*). In particular, *TP53* is the most frequently mutated tumor suppressor gene in HNC, affecting 72% of HNC patients (365/510 cases) and 76% of HNC oral cancer ones (239/313). By mutation mapping, we found that both AT-84 and SCC VII carried the *Trp53* p.Arg270 hotspot mutation (equivalent to the well-known oncogenic human *TP53* p.Arg273 hotspot mutation), while 4MOSC2 carried *Trp53* p.Glu221 and p.Gly241 mutations, which are equivalent to human *TP53* p.Glu224 and p.Gly244 hotspot mutations found in HNC patient tumors (Figure 2D). Specifically, for the oncogenic *TP53* p.Arg273C [54], AT-84 and SCC VII harbored this mouse counterpart mutation with high allele frequencies of 1.0 and 0.6, respectively. This is consistent with TCGA findings in HNC tumors that *TP53* mutations are key driver events for most HNC tumors.

### 2.3. Aberrations of Immune Evasion and Epigenetic Machineries, the Notch, DNA Repair, and Receptor Tyrosine Kinase and TGF-β/Smad4 Pathways are Common

**Figure 3:**
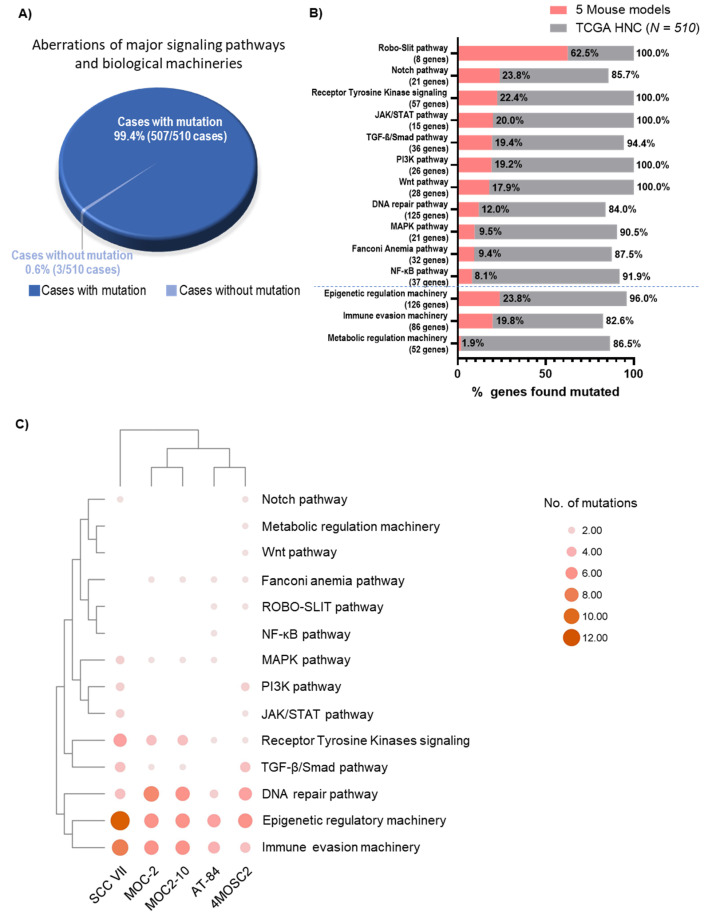
Mouse models with HNC-relevant genetic aberrations of major signaling pathways and biological machineries. (**A**) Pie chart showing percentages of patient tumors harboring mutations of 14 pathways and machineries (Pathways: PI3K, MAPK, JAK/STAT, Notch, Wnt, NF-κB, TGF-β/Smad, ROBO–SLIT, Fanconi anemia, receptor tyrosine kinases; Machineries: DNA repair, immune evasion, metabolic regulation, epigenetic regulation) in TCGA-HNC (*N* = 510) with the 3D sector representing mutations of the same genes found in 5 mouse models. (**B**) Bar graph showing the number of pathway/machinery genes mutated in TCGA-HNC (*N* = 510) (grey bars) and the 5 mouse models (red bars). (**C**) Bubble plot showing the abundance of TCGA-HNC (*N* = 510) as well as 14 pathways and machineries’ mutations across 5 mouse models (AT-84, SCCVII, MOC-2, MOC2-10, and 4MOSC2).

Our recent signaling pathway analysis revealed that mutations of 7 major signaling pathways affect over 85% of HNC tumors (including the MAPK, PI3K, JAK/STAT, NF-κB, Notch, Wnt, and TGF-β/Smad4 pathways) [19]. Here, with the further inclusion of 4 additional signaling pathways (the receptor tyrosine kinase (RTK) signaling, DNA repair, Fanconi anemia (FA), and ROBO–SLIT pathways) and 3 key biological machineries relevant for HNC tumorigenesis (immune evasion, epigenetic, and metabolic regulatory machineries), we found that nearly all TCGA-HNC tumors (99.4%, 507/510) harbored mutations of at least one of the 14 pathway/machinery genes (Figure 3A; Table S5). Among all 14 HNC-relevant pathway/machineries, we found that the ROBO–SLIT pathway aberrations are most represented by these 5 mouse models, with 62.5% of the ROBO–SLIT pathway genes altered (5/8 genes). These include the *Robo1*, *Robo2*, *Robo3*, *Robo4*, and *Slit1* genes. This is followed by aberrations of the Notch, RTK signaling, JAK/STAT, TGF-β/Smad4, PI3K, Wnt, DNA repair, MAPK, FA, and NF-κB pathways, as well as the epigenetic, immune evasion, and metabolic machineries. Although few, these 5 mouse models do appear to capture HNC patient tumors’ diversified pathway aberrations (Figure 3B). Notably, all 5 models are somatically altered in immune evasion and epigenetic machineries, as well as in the DNA repair and RTK pathways, consistent with the known mechanisms of HNC tumorigenesis. The well-known carcinogenic pathways, TGF-β/Smad4, MAPK, and FA, are also mutated in 4 mouse models (Figure 3C). Lastly, SCC VII and 4MOSC2 have the highest number of somatic mutations across most of the pathways or machineries, consistent with their high mutation rates.

### 2.4. ERBB4 and KRAS Mutations Are Frequently Captured by Metastatic Mouse Models and Metastatic HNC Tumors

Precision drugging of invasive and metastatic HNC remains largely unexplored. Given the emerging roles of immunity and host inflammation in cancer metastasis and disease progression, these immunocompetent metastatic models would be invaluable research tools. In order to assess the potential utilities of these 5 immunocompetent metastatic HNC mouse models for HNC metastasis research, we first assembled an “HNC invasion/metastatic candidate genome database”, and then compared candidate invasion/metastatic events between TCGA-HNC tumors and these 5 models. The database collected genes from the cancer hallmark “activating invasion and metastasis” gene set [52], as well as genes with demonstrated experimental evidence [55,56,57,58,59] (manually curated) or from the GeneRIF bioinformatics resource (https://www.ncbi.nlm.nih.gov/gene/about-generif/; with keyword filtering of “metastasis”, “HNSCC”, “OPSCC”, “OSCC”, etc.). The compiled “HNC invasion/metastatic candidate genome database” comprises a total of 1283 human genes, and their corresponding mouse gene names are also listed for referencing (Table S6).

**Figure 4:**
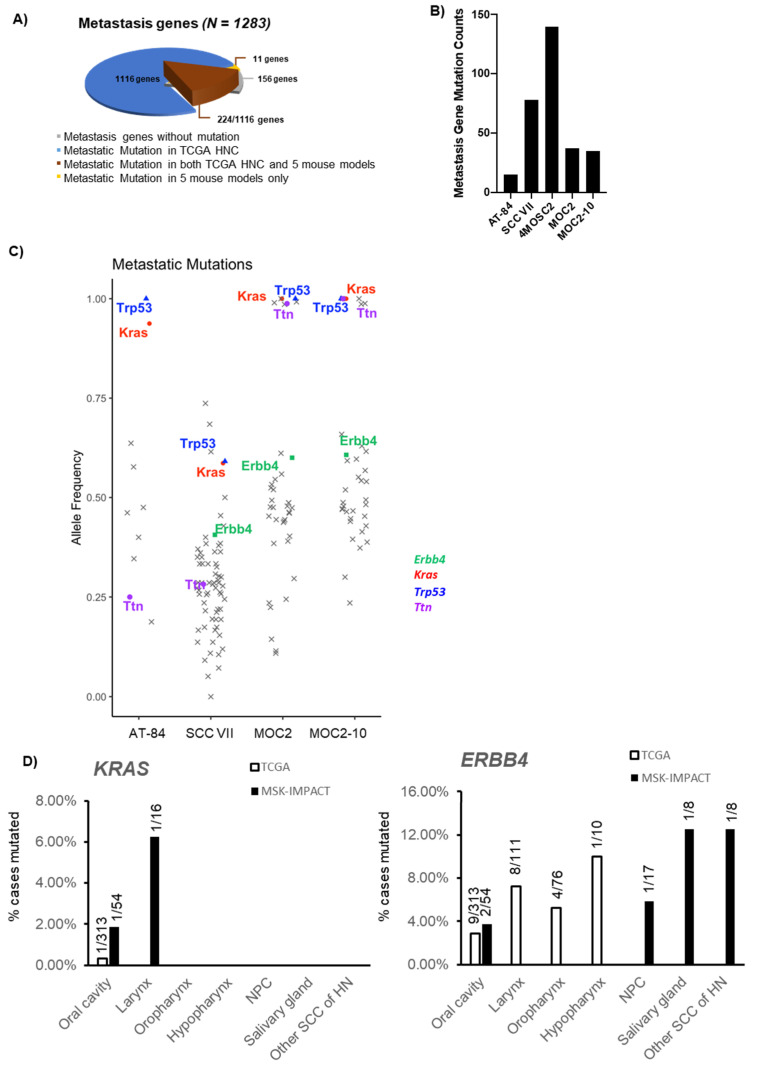
Genetic analysis of 5 mouse models reveals metastasis-related alterations. (**A**) Pie chart showing the number of metastasis-related genes altered in 5 mouse models vs. TCGA-HNC patient tumors (*N* = 510). (**B**) Bar graph showing somatic mutation counts of metastasis-related genes in each of the five mouse models. (**C**) Scatter plot showing allele frequencies of metastasis-related gene mutations in each mouse model. Mutations harbored by all 5 mouse cell lines or highly related to cancer metastasis are highlighted with different marks, as displayed on the right. (**D**) Bar graph showing the mutation rates of *KRAS* and *ERBB4* in HNC patient tumors from the TCGA cohort (white bars) vs. the metastatic HNC from the Memorial Sloan Kettering Cancer Center (MSK)-IMPACT cohort (black bars). Detailed subsites are shown. NPC: nasopharyngeal carcinoma; SCC: squamous cell carcinoma; HN: head and neck.

Collectively, TCGA-HNC patient tumors carried somatic mutations of 1116 of the 1283 metastatic candidate genes, while the 5 mouse models harbored mutations of 235 of such genes, among which 11 were not found to be mutated in HNC patient tumors (Figure 4A, Tables S7 and S8). With the 235 candidate genes found mutated in the mouse models, 132 (56.2%) of them have never before been studied in HNC. The distribution of mutation counts on the 235 genes in each of the five models is shown in Figure 4B, with 4MOSC2 carrying the largest number of mutations of these genes, consistent with their overall high mutation counts in general (Table S8). For copy number changes, 1156 of the 1283 metastatic candidate genes are altered in HNC patient tumors, while 471 of them are altered in AT-84 and SCC VII (Tables S7 and S8).

Interestingly, among the 4 mouse models with detailed allele frequencies, we noted that *Kras* and *Erbb4* genes were mutated in 4/4 and 3/4 models with high allele frequencies ranging from ~0.4 to 1.0, respectively (Figure 4C, Table S8, allele frequency data of 4MOSC2 not available). These events have not been previously noted in HNC patient tumors [53]. This is especially the case for *Kras* mutations, which appear to be uncommon in the TCGA-HNC cohort (0.2%; 1 of 510 patients), but were found to be mutated in as high as 15% (1643/10,945 patients) of advanced/metastatic solid tumors and 1.07% (2/186 patients) in metastatic cancer annotated as HNC in the MSK-IMPACT cohort from the Memorial Sloan Kettering (MSK) Cancer Center [60]. This unexpected relatively higher rate of *KRAS* mutations in HNC patient tumors implicates their likely causative role in HNC metastasis, which can potentially be druggable. Subsite analysis showed that, in oral cavity cancer, *KRAS* mutations occur in 0.32% in TCGA-HNC but 1.85% in MSK-IMPACT (Figure 4D). Similarly, the metastatic gene *Erbb4* was found to be mutated at a slightly increased rate of 3.70% in MSK-IMPACT oral cavity cancer vs. 2.88% in TCGA-HNC oral cavity cancer (as well as notable *Erbb4* mutations in 5.88% (1/17 cases) of nasopharyngeal cancer, 12.50% (1/8 cases) of salivary gland cancer, 12.50% (1/8 cases) of other squamous cell carcinomas (SCCs) of the head and neck, and 11.11% (1/9 cases) of cancers of unknown primary in the head and neck region, as annotated by MSK-IMPACT (https://www.cbioportal.org/study/summary?id=msk_impact_2017; Table S9). Note that subsite classification showed that in the TCGA-HNC dataset, besides oral cavity cancer, cancers of the laryngeal, oropharyngeal, and hypopharyngeal subsites also have *Erbb4* mutations, but absent in MSK-IMPACT advanced/metastatic cancers of these subsites (Table S9). Specifically, *Erbb4* p.F1071S and p.E1168* were found in 3 of the metastatic mouse models.

### 2.5. The Models Carried Aberrations of a Wide Spectrum of Immunomodulatory Molecules and Chemokine Signaling Events

**Figure 5:**
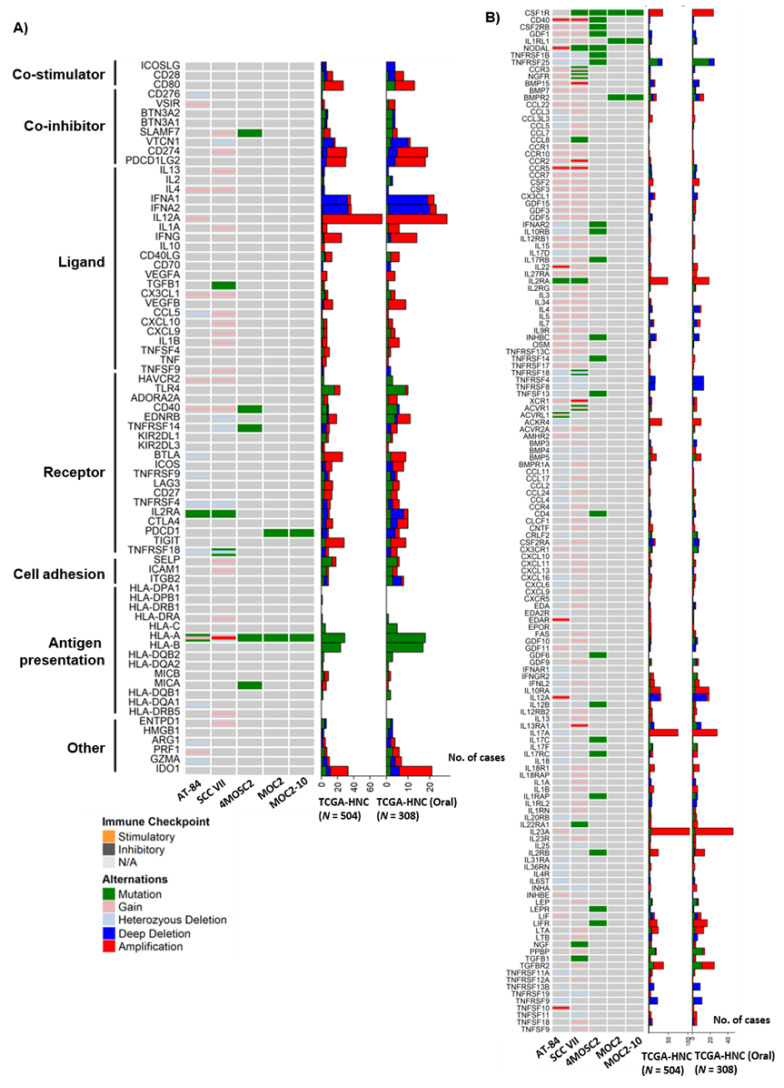
Genetic aberrations of immune-related genes in 5 mouse models vs. HNC patient tumors (*N* = 510). (**A**) Oncoprints showing mutational and copy number alterations of immunomodulators of 5 mouse models vs. TCGA-HNC patient tumors as well as TCGA-HNC oral tumors. (**B**) Oncoprints showing mutational and copy number alterations of cytokine–cytokine interaction molecules in 5 mouse models and corresponding genetic aberrations in TCGA-HNC patient tumors and TCGA-HNC oral tumors.

In order to employ or to allow rational engineering of these models to address specific immune-related questions, their immune profiles must first be defined. First, we examined if these models carry any intrinsic aberrations of a wide spectrum of immunomodulatory molecules, which have the ability to stimulate or suppress cancer growth when targeted by immunomodulatory drugs, theoretically. These immunomodulatory molecules included 75 genes involved in antigen presentation, inhibitory and stimulatory immune checkpoint molecules defined by Thorsson et al. [6]. The human-to-mouse gene name conversions are listed in Table S10. As shown in Figure 5A, 4MOSC2 and SCC VII harbored the most somatic mutations of these genes. Specifically, 4MOSC2 has mutations of two MHC Class I/II genes, namely *H2-K1* (equivalent to human *HLA-A* gene) and *Mill1* (*MICA*), which are involved in antigen presentation, as well as an array of inhibitory and stimulatory immune checkpoint molecules, including *Slamf7* (*SLAMF7*), *Cd40* (*CD40*), and *Tnfrsf14* (*TNFRSF14*). For *PD-1/PD-L1*, MOC2 and MOC2-10 have *Pdcd1* (*PDCD1*, *PD-1*) mutations, whereas AT-84 carried an *Il2ra* (*IL2RA*) mutation. Although gene copy changes were only available for AT-84 and SCC VII per our exome sequencing, we did find that these 2 models could partially capture gene copy changes of some immunomodulatory genes as noted in the TCGA-HNC tumors (Figure 5A). These include a copy number increase of *Cd274* (*CD274*), and losses of tumor necrosis factor receptor (TNFR) superfamily genes, including *Tnfrsf 18* (*TNFRSF18*).

Within the immunomodulating gene set defined by Thorsson et al. [6], there are 27 cytokine/cytokine receptors. We noted that SCC VII carried mutations or copy number changes in a number of these genes, including *Il2ra* (*IL2RA*) mutation, copy number gains of *Ccl5* (*CCL5*), *Cx3cl1* (*CX3CL1*), *Cxcl9* (*CXCL9*), *Cxcl10* (*CXCL10*), *Il1a* (*IL1A*), *Il1b* (*IL1B*), *Il4* (*IL4*), and *Il13* (*IL13*), suggestive of altered chemokine signaling in SCC VII. In contrast AT-84 carried an *Il2ra* (*IL2RA*) mutation, as well as copy number gains of *Cx3cl1* (*CX3CL1*), *Il4* (*IL4*), and *Il12a* (*lL12A*), and heterozygous loss of *Ccl5* (*CCL5*). Collectively, these 5 models encompass diverse immunomodulatory aberrations partially representing the heterogeneous immunomodulatory signaling of HNC.

Given the importance of cytokine signaling in HNC biology, we further comprehensively analyzed all cytokine defects carried by the 5 models, as only a small subset of cytokine signaling molecules were included in Thorsson’s gene list (which was mainly confined to potentially druggable immunomodulatory events [6]). As shown in Figure 5B, among all 159 cytokine–cytokine receptor interaction events (Kyoto Encyclopedia of Genes and Genomes, KEGG, https://www.genome.jp/kegg/, hsa04060), we found that 4MOSC2 carried the highest number of mutations (22/159) in these cytokine-related genes, followed by SCC VII (10/159 genes), MOC2 (4/159), MOC2-10 (4/159), and AT-84 (2/159) (Figure 5B). The colony-stimulating factor 1 receptor (*Csf1r*), which promotes survival of hematopoietic precursor cells, was found to be mutated in 4/5 mouse models (except for AT-84). Regarding AT-84 and SCC VII with CNV data available for analysis, we found that AT-84 harbored gene copy losses of 49 of these genes, including genes of the C-C motif chemokine (CC) subfamily, the C-X-C motif chemokine (CXC) family, the TNF family, and the interleukin (IL) family. Based on the gene-level copy changes, we found that SCC VII harbored copy number gains/amplifications of 85 amplifications of genes across the CC chemokine subfamily, the CXC chemokine family, the TNF family, the interleukin (IL) family, and the TGF-β family (Figure 5B).

### 2.6. ERBB4, RTKs, MAPK Pathway, DNA Damage and Cell Cycle Pathways Are Druggable Events Represented by These Models

**Figure 6:**
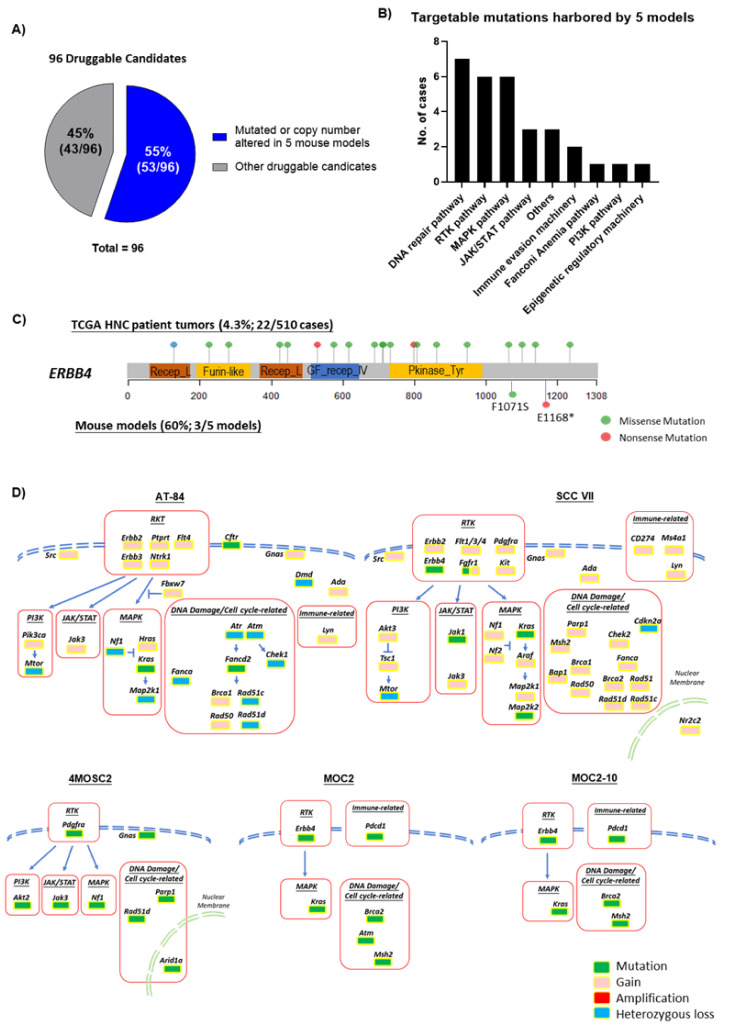
Potential druggability of genetic events represented by 5 mouse models vs. HNC patient tumors (*N* = 510). (**A**) Pie chart showing 55% (53/96 genes) of druggable candidates are mutated or copy number altered in 5 mouse models. (**B**) Pie chart showing 13% of druggable events harbored by 5 metastatic mouse models are genes in the RTK signaling, followed by DNA repair pathway, other unclassified mutations, MAPK pathway, FA pathway, PI3K pathway, immune evasion machinery, JAK/STAT pathway, epigenetic regulatory machinery, and Notch pathway. (**C**) Mapping of mutation sites of ERBB4 represented by HNC patient tumors (upper) and the 5 mouse models in the tyrosine kinase domain (lower). (**D**) Summary mapping of aberrations of druggable candidates in 5 mouse models based on the RTK, MAPK, PI3K, NOTCH, JAK/STAT, and DNA damage/cell cycle-related pathways.

Using the most updated druggable gene list including 96 genes currently or potentially druggable by FDA-approved drug indications or potential agents (Table S11), we examined the current druggability of HNC patient tumors and these 5 models. As shown in Figure 6A, it appears that these 5 models have 53/96 (55%; Table S12) druggable candidates mutated or CNV-altered, whereas TCGA-HNC tumors carried the mutations of the majority of these 96 druggable candidates (93/96; 96.9%; Table S13). Figure 6B shows the distribution of drug candidates that are mutated in these 5 models (ranked from the highest to the lowest), spanning the DNA repair pathway, receptor tyrosine kinase pathway, MAPK pathway, JAK/STAT pathway, others, immune evasion machinery, FA pathway, PI3K pathway, and epigenetic regulation machinery. Among the 58 receptor tyrosine kinases (RTKs) of the entire human RT kinome, we found that these 5 models carried a total of 21 mutations of 13 RTKs, namely *Erbb4*, *Ephx1*, *Ltk*, *Fgfr1*, *Epha6*, *Ddr1*, *Csf1r*, *Tyro3*, *Ror2*, *Epha4*, *Pdgfra*, *Lmtk3*, and *Insr* (Figure 6C). Importantly, *Erbb4* mutation was commonly identified in 3 out of 5 models (p.F1071S and p.E1168*) (Figure 6C). Among the druggable targets, we also note that *Pdcd1*, *Jak1*, *Hras*, *and Map2k2* are mutated, thus respective inhibitors could potentially be tested with these models to determine if these aberrations could confer sensitivity to respective PD-L1 inhibitors, JAK/STAT, MEK, and RAS inhibitors, or not. Lastly, based on these findings, we summarized a druggable gene map for each of the five models (Figure 6D, with detailed gene list in Table S12). In brief, the RTK gene family, MAPK pathway (mainly *Kras*), DNA damage and cell cycle gene aberrations are found altered in all 5 models, whereas JAK/STAT aberrations are only found in AT-84, SCC VII, and 4MOSC2.

## 3. Discussions

As of today, there are five convenient xenograftable immunocompetent metastatic HNC models. Our whole-exome analyses of the two most commonly used models, namely AT-84 and SCC VII, reveal mutational signatures of APOBEC edit, tobacco smoke, UV damage, aging-related signature, and microsatellite instability, similar to that of HNC patient tumors. Importantly, extensive evaluations of the human HNC relevance of all five immunocompetent metastatic HNC models demonstrated mutational hallmarks representative of sustaining proliferation, invasion/metastasis, and resisting cell death, consistent with the reported metastatic nature of these models. Cancer gene analyses indicate the presence of several candidates that have not been previously investigated in HNC. Metastatic gene analyses also uncover new metastasis candidate events that have not been previously examined in HNC, including *Muc5ac*, *Trem3*, *Ttn*, and many others. Strikingly, *Erbb4* and *Kras* mutations are present in HNC metastases with relatively higher mutation frequency than in primary oral tumors. Our results first emphasize that as few as five metastatic cell models, potential key human-relevant HNC metastatic aberrations, are being frequently recapitulated. Thus, these models can be readily utilized for metastasis biology studies or drugging of metastatic genetic aberrations, which is an area of emerging importance in HNC in this post-genome era. A summary in Table S14 lists key differences and similarities among these five mouse models for easy referencing and utility determination for the research community.

In terms of immunoregulatory molecules and cytokine/chemokine signaling, the five models partially harbor diverse genetic events as in human HNC tumors. These findings not only imply that more models are to be established to better mimic patient tumors in terms of immunoregulation, but also ultimately define which of these models can be amenable for genetic manipulation if immune regulatory genes and host immune interactions are to be examined one by one in vivo. Lastly, our druggability analyses demonstrate that with these five models alone, they seem to provide a sizeable list of druggable mutations for precision medicine developments, with a decent number of druggable events for metastatic candidates, DNA repair, receptor tyrosine kinase, as well as the JAK/STAT and MAPK/RAS pathways. Our results not only inform the human HNC relevance of these models, but also provide an extensive catalogue of genetic events for both basic and translational HNC research. The community can reference our results to choose the right models for the study of endogenous aberrations, or choose a suitable model based on their genetic background delineated in this study for engineering a specific candidate gene in question. Nevertheless, one has to bear in mind that there are some limitations of these mouse immunocompetent models, which are universal for any tumor types, including HNC. First, despite our detailed genetic characterizations, these tumor models were derived from mouse origin, but not human. Therefore, there could be biological differences between the gene or mutation functions in mouse vs. human. Second, these mouse xenografts are surrounded and supported by mouse stroma; thus, the study of tumor genetic events on stromal interactions will only be relevant in mouse contexts, including immune interactions. Third, therapeutically and for examples, the mouse version of antibodies may be needed for efficacy determination, such as the mouse anti-PD-L1 antibody. Any therapeutic results generated from mouse would still need to be fully investigated in human clinical trials with human versions of the therapeutic antibodies, for example. It should be noted that more sophisticated models, such as human HNC patient-derived xenograft (PDX) models, or even humanized PDX models are possible alternatives for the study of human somatic mutations on tumor growth, drug responses, as well as human immune cell interactions. Yet, these PDX models have to be generated with great coordinated clinical–preclinical research efforts and genomically characterized, and may not be of easy or equal access for experimentation for all investigators at this moment.

In conclusion, our findings first highlight the HNC clinical relevance of these immunocompetent metastatic HNC models that may allow future drugging studies or biology studies to be conducted in host immune contexts. Lastly, with only five such models, a number of previously novel metastasis-related and drug targets are revealed for plausible precision medicine investigations. More such models are worth establishing for biology hypothesis-driven studies as well as treatment explorations.

## 4. Materials and Methods

### 4.1. Cell Lines

The AT-84 cell line was a kind gift from Dr. Michael P. Hier (Department of Otolaryngology-Head and Neck Surgery, Jewish General Hospital, Montreal, QC, Canada). SCC VII was kindly provided by Professor Sven Brandau (Department of Otorhinolaryngology, University Hospital Essen, Essen, Germany) and Dr. Herman D. Suit (Department of Radiation Oncology, Harvard Medical School/Massachusetts General Hospital, Boston, MA, USA). Both AT-84 and SCC VII were cultured in RPMI medium 1640 (Thermo Fisher Scientific, Waltham, MA, USA) with 10% heat-inactivated fetal bovine serum (FBS) and 1× penicillin (100 units/mL)/streptomycin (100 μg/mL) (Thermo Fisher Scientific). Cells were maintained in a humidified incubator (Forma™ Series II 3110 Water-Jacketed CO2 Incubator, Thermo Fisher Scientific, Waltham, MA, USA) of 5% CO_2_ at 37 °C.

### 4.2. Exome Sequencing

Normal DNA was extracted from the tail of C3H mouse (Augusta University, Augusta, GA, USA). DNA from AT-84 and SCC VII were extracted using the Qiagen DNAeasy blood & tissue kit (Qiagen, Hilden, Germany) as per the manufacturer’s protocol. DNA samples (a minimum of 200 ng) were whole-exome sequenced by DNA link (DNA Link, Inc., Seodaemun-gu Seoul, Korea) using the Illumina Novaseq 6000 platform (Illumina, San Diego, CA, USA) with ~50× coverage. Raw FASTQ files were processed for further analysis using GATK tools (Broad Institute, Cambridge, MA, USA). The FASTQ files of AT-84 model are archived in doi: 10.6084/m9.figshare.12623786 and the FASTQ files of SCC VII model are archived in doi: 10.6084/m9.figshare.12623882.

### 4.3. Mutation and Copy Number Calling

Somatic mutations were detected by the matched tumor/normal pair through Mutect (version 1.1.7, Broad Institute, Cambridge, MA, USA) and Strelka (version 1.0.14, Illumina, San Diego, CA, USA). Mutation profile and COSMIC mutation signature were called used R package “MutationalPatterns” (Version 2.0.0). Copy number analysis was performed using CNVkit (version 0.8.5; https://cnvkit.readthedocs.io/en/stable/). Instead of using the matched normal data from each sample, we pooled entire normal samples’ data set together to calculate the log2 copy ratio for individual bins and segments. Scatter plot shows bin-level log2 coverages and segmentation calls together. Chromosome plots display segment gain or loss and corresponding genes in chromosome scale.

### 4.4. Databases

The cancer hallmark gene sets were previously defined by Zhang et al. [52]. The cytokine–cytokine receptor interaction gene set was downloaded from the Kyoto Encyclopedia of Genes and Genomes database (KEGG, hsa04060).

## 5. Conclusions

Comprehensive genomic analyses of these current five xenograftable models have informed us about new potential drug targets for metastatic HNC. Immunocompetent metastatic HNC models are worth developing to address therapy- and invasion/metastasis-related questions in host immunity contexts.

## Supplementary Materials

The following are available online at https://www.mdpi.com/2072-6694/12/10/2935/s1, Figure S1: Oncoprints showing respective CNVs of *ROBO2*, *FANCB*, *EGFR*, *PIK3CA*, *FBXW7*, *CDKN2A*, *FANCF*, *AJUBA*, *RB1*, and *ATR* genes in TCGA-HNC (*N* = 510), Table S1: List of non-synonymous somatic mutations of AT-84 and SCC VII, Table S2: List of copy number variations of AT-84 and SCC VII, Table S3: List of 830 cancer genes, Table S4: Genetic aberrations of 830 cancer genes in each mouse model, Table S5: Definitions of 11 major signaling pathways and 3 biological machineries, Table S6: Metastatic gene list of 1283 metastasis-related genes, Table S7: Genetic aberrations of 1116 metastatic genes and copy number change in 1156 metastatic genes in TCGA-HNC patients (*N* = 510), Table S8: Genetic aberrations of 149 metastatic genes and copy number change in 894 metastatic genes in 5 mouse models, Table S9: Comparison of TCGA-HNC and IMPACT-MSK head and neck cancer subsites as well as *KRAS*/*ERBB4* mutations. Table S10: List of immunomodulatory genes and annotations, Table S11: List of druggable candidate genes, Table S12: Potential druggable genetic events in each of the 5 mouse models, Table S13: Potential druggable genetic events in TCGA-HNC (*N* = 510). Table S14: Summary of key similarities and differences in genetic events including metastasis-related genes, immunomodulatory genes, and potential druggable events and corresponding therapeutic drugs for easy referencing and utility determination.
