# Supplementary material for: Comprehensive Exome Analysis of Immunocompetent Metastatic Head and Neck Cancer Models Reveals Patient Relevant Landscapes
Source: Cancers (Basel). 2020 Oct 12;12(10):2935. doi: 10.3390/cancers12102935 (PMC7601118; doi:10.3390/cancers12102935)
Supplement: Supplementary file 1 [file cancers-12-02935-s001.zip › cancers-964870 supplementary figures.pdf]

cancers

Article

Comprehensive Exome Analysis of Immunocompetent Metastatic Head and Neck Cancer Models Reveals Patient Relevant Landscapes

Hui Li, Hoi-Lam Ngan, Yuchen Liu, Helen Hoi Yin Chan, Peony Hiu Yan Poon, Chun Kit Yeung, Yibing Peng, Wai Yip Lam, Benjamin Xiaoyi Li, Yukai He and Vivian Wai Yan Lui

Supplementary Materials

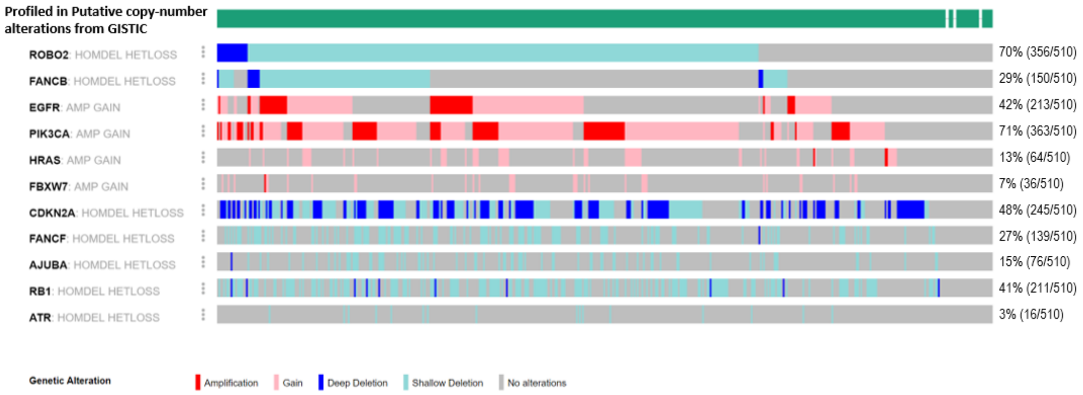

Figure S1. Oncoprints showing respective CNVs of *ROBO2*, *FANCB*, *EGFR*, *PIK3CA*, *FBXW7*, *CDKN2A*, *FANCF*, *AJUBA*, *RB1* and *ATR* genes in TCGA-HNC (*N* = 510).

© 2020 by the authors. Licensee MDPI, Basel, Switzerland. This article is an open access article distributed under the terms and conditions of the Creative Commons Attribution (CC BY) license (http://creativecommons.org/licenses/by/4.0/).
